# On the Behavior of Alcohol in the Animal Organism

**Published:** 1854-02

**Authors:** 


### *On the Behavior of Alcohol in the Animal Organism.* By Dr. Ducheck. (Prag. Vjhrschr., X. 3, 1853.)

(Translated for the Examiner.)

There are now six kinds of alcohol well known, the æthyl, methyl, amyl, and acryle alcohols, the cetyle and the glyceryle. To each of these alcohols, possessing the general formula ($C_nH_x + O + HO$), there is a corresponding aldehyde ( ($C_nH_x — H_2$)$+ O + HO$) which differs from the alcohol by possessing two equiv. less of hydrogen; and also a group of characteristic acids having the formula ($C_nH_x — H_2 + O_3 + HO$). Each of these acids contains two equiv. more of oxygen than its corresponding aldehyde. To the æthyl-alcohol belong the acetal-aldehyde and the acetic acid; to the methyl-alcohol, the formic aldehyde and the formic acid; to the amyl-alcohol, the amyl aldhyde and the valerianic acid, &c., &c.

In order to change the alcohol into aldehyde, two equivalents of oxygen are absorbed, forming two equivalents of water, then the aldehyd again absorbs two equivalents more, forming water and acetic acid.

Alcohol $C_4H_6O_2 + O_2 = 2HO + C_4H_4O_2$ aldehyde.

Aldehyde $C_4H_4O_2 + O_2 = 2HO + C_4H_3O_3$ acetic acid.

The process of oxidation, however, does not end here:

Acetic acid $C_4H_3O_3 + O = HO + 2C_2HO_3$ formic acid.

Formic acid $C_2HO_3 + O = HO + C_2O_3$ oxalic acid.

Oxalic acid $C_2O_3 + O = 2C\ O_2$, carbonic acid.

It is apparent from this, that the process of the transformations of alcohol takes place by the continued absorption of oxygen, whereby finally water and carbonic acid are formed. The process of oxidation in the other kinds of alcohol is the same as in the æthyl alcohol.

The author, by means of a stomach pump, introduced into the stomachs of three dogs large quantities of absolute alcohol (from half ℥ to 60 grammes,) partly pure and partly diluted; he also injected into the rectum of a fourth dog 1½ ʒ of fusel oil diluted with water. The phenomena exhibited in all the four cases were, 1. Intoxication more or less strong, and death sooner or later, according to the quantity of alcohol introduced, and to the slow or rapid manner of administration. 2. These phenomena occurred in the same manner whether the alcohol had been introduced into the stomach or the rectum. 3. The blood appeared somewhat darker, was of an alkaline reaction, and was distinguished by an odor like aldehyde, that was diffused through all the organs. 4. In two cases aldehyde was detected in the blood; while 5th, no further product of oxidation of the same was found. 6. Alcohol was not once detected in the blood. 7. The stomach, even a short time after the introduction of alcohol, contained only a very small amount of it. 8. The urine, as well as the fluid in the cerebral ventricles, exhibited a peculiar but evanescent odor of ether. 9. No important anatomical change existed in any of the organs. 10. The action of amyl-alcohol appeared far more decided than that of the æthyl-alcohol. From these facts it follows: that the alcohol becomes absorbed and chemically changed, and that these two actions occur so rapidly, that even after a very short time we can only detect the aldehyde. The impossibility of detecting alcohol in the blood is opposed to the prevailing view, that alcohol in an unchanged atomic combination remains for a short time in the blood. This view rests upon the observation that, after the use of a large quantity of spirituous liquors, the breath is charged with a kind of alcoholic odor, or rather that peculiar to the drink that has been taken; and also that in the opening of the bodies of those who have suddenly died, an odor of alcohol is diffused from the inner organs. These cases, the author considers, are all founded upon incorrect observation. In dissection the odor of alcohol is confounded with that of aldehyde, and as for the breath, it carries along with it, besides the peculiar pulmonary exhalations, that portion of the liquor that remains in the form of vapor in the cavities of the mouth and throat. Other substances besides aldehyde are contained in the breath, and exhaled undecomposed at the same time, such as the peculiar aromas of the various liquors, which can be distinguished from the pulmonary exhalations. They originate from the various substances associated with the alcohol, viz., the different kinds of ether; the œnanthic ether,

(from wine and cognac,) the butyric ether, from rum, and also ethereal oils, various acids, &c. The odors of these substances are generally characteristic of the specific drink, and they are identified with that of alcohol, and it is then assumed that this is exhaled, and therefore remains undecomposed in the blood. If it be granted that alcohol is oxydized in the system into aldehyde, then it must be admitted that this oxidation takes place either in the stomach or in the blood. Oxygen is, in every case, necessary to the transformation of the alcohol; in this respect, therefore, the formation of aldehyde meets with no impediment in these places. The change, however, cannot take place in the stomach, since, 1st, we can never detect the presence of aldehyde in it; and 2d, since the conditions requisite to the formation of aldehyde in the stomach are the same as in atmospheric air, the change of the alcohol in the stomach taking place slowly, as when exposing a large amount of it to the atmosphere, so that intoxication would occur much later. This view will finally appear untenable, if we but consider that intoxication, consequently the formation of aldehyde, occurs equally if the alcohol be introduced into the rectum, whose cavity, as is well known, contains no oxygen. If now the alcohol remains unchanged in the stomach, its transformation must necessarily take place in the blood. The question arises, how does the alcohol enter the blood? Many experiments which the author made of the penetrability of the membranes (hog's bladder) to alcohol and aldehyde, place it beyond doubt that the walls of the stomach can be penetrated by the alcohol, and thus be taken up by the vessels. Another question now arises; does the transformation of the alcohol take place after it has been carried unchanged to a remote part of the body, or immediately after its absorption by the blood vessels? Several facts are opposed to the first supposition. Whenever alcohol in great amount comes in contact with blood, coagulation necessarily occurs. This is proved by the daily experience and unsuccessful attempts of earlier experimenters, who have studied its action whilst injecting alcohol in the veins. Such coagulation will then occur if, as in the first three experiments of the author, a great quantity of alcohol penetrates through the walls of the stomach without oxydizing, that is to say, without becoming changed into aldehyde. And yet in the bodies of men or animals that have suddenly died from intoxication, there has never been found any trace of coagulation, that had taken place during life. Experiments show that only a very large amount of aldehyde, introduced rapidly and in an undiluted condition into the veins, can produce coagulation; that this, therefore, never takes place when introduced slowly in small quantities and tolerably diluted. From this, therefore, we conclude, that alcohol only after it has been changed into aldehyde can remain any length of time in the blood. Besides the rapid penetration of aldehyde into all the tissues even in one or two minutes, can be more readily explained by its greater capacity of imbibition, than by assuming the same of alcohol, and supposing the change into aldehyde to take place only after the imbibition of the former into the organs.

These reasons and the impossibility of chemically detecting alcohol in the blood, make it probable that the alcohol enters only the absorbent vessels, and that on the appearance of even the smallest amount in the interior of these, oxidation immediately follows. This view of the rapid formation of aldehyde is supported by the large surface and fine porous condition of the walls of the stomach. Since then it is assumed that intoxication is not produced by the action of alcohol upon the stomach, it must therefore commence with the formation of aldehyde and this consequently must be the intoxicating principle. The author to prove this, injected into the veins of three and into the stomach of two dogs, aldehyde partly diluted and partly undiluted. He obtained these results. 1. That aldehyde introduced into the venous blood or stomach, produces the same violent symptoms of intoxication as if alcohol were used. 2. The sudden introduction of a large amount of aldehyde into a blood vessel, produced mechanical coagulation of the blood. 3. After the passing off of the narcotic effect, acetic and oxalic acids were found in the blood; from this it appears that aldehyde is fitted for excretion by the absorption of oxygen. 4. If there is at any one moment a large amount of aldehyde in the blood, a portion of it can be thrown off unchanged, with the exhalations from the lungs. Hence arises the odor of the breath, that has been confounded with that of alcohol. This symptom only appeared when a great amount of aldehyde had been rapidly injected. The lengthened respiration, indicated the necessity for oxygen, the animal heat appeared to keep parallel with the increased process of combustion.

If it now be granted that intoxication commences with the formation of aldehyde, it may be asked whether it ends with its disappearance from the blood, or whether the symptoms of intoxication are connected with the presence of the further process of oxydation, acetic acid, &c. The author's experiments show that acetic acid is never present in the blood of animals that have died during intoxication, although it has been found after this has passed off. Besides, the author made the direct experiment of injecting dilute acetic acid into the veins of a dog—no narcotic effects followed. These facts lead to the conclusion that the period of intoxication is limited to the disappearance of the aldehyde, that is to say, to its conversion into acetic acid. Consequently the above stated view, that intoxication is due properly only to the aldehyde receives further confirmation. In two experiments oxalic acid was found in the blood, as a further product of the oxidation of alcohol. The ultimate products of this continued oxidation must of course be carbonic acid and water. For the determination of carbonic acid and water, in the pulmonary exhalations, after the use of alcohol, the author made three experiments upon dogs, in which at the same time, the amount of expired air, the animal heat, the number of respirations, and arterial pulsations were taken into account. He obtained the following results: 1. After the use of alcohol more air is inhaled, indicating an increased necessity for oxygen. 2. In the expired air is found (with equal amount and equal number of inspirations) *less carbonic acid and less water* in the pulmonary exhalations. (A result which Vierordt and Böcker also obtained.) 3. The number of respirations and arterial pulsations, together with the animal heat, consequently the process of oxidation, were greatly augmented.

When the process of alcoholic combustion by the continued absorption of oxygen takes place, the blood is in this way deprived of a great amount of oxygen, which would otherwise be applied to the combustion of other substances. The blood therefore must undergo a change, and the normal course of metamorphoses be impeded; and the quantity of the final products—carbonic acid and water—also must vary. In the normal condition

(besides other substances) grape sugar is consumed in the blood, and its transformations into carbonic acid and water depend upon oxidation, a process of great importance to respiration and the generation of heat. If a substance though, like aldehyde, enters the blood, making energetic demands upon oxygen, and generally having a greater affinity for it than sugar has, then the *latter must in greater or less amount remain unconsumed,* at least for a time. That this actually takes place, the author proves by his investigations of cases of sudden death by alcohol and aldehyde, as well as by long continued use of alcohol, in which cases, he always found a large quantity of sugar in the blood, and, indeed, larger than in the blood of two dogs drawn in the course of the experiment, *before* the first intoxication. If alcohol ($C_4 H_6 O_2$) is burnt, the proportion of carbonic acid to water is as 4 : 6 or 1 : 1½ ; in the combustion of sugar ($C_{12} H_{12} O_2$) as 1 : 1. In the first case therefore there is more water and less carbonic acid formed. If now, whilst using alcohol, it alone is consumed, the sugar remaining unchanged in the blood, then the proportion of carbonic acid to water that is expired, is changed, and this is the reason why *after the use of alcohol* less carbonic acid is expired. Concerning the second product of combustion—water—in spite of its outweighing the carbonic acid, its amounts are very inconsiderable. Since now the kidneys are especially designed for the secretion of water, then it is evident why its amount is greatly decreased in the exhalations from the lungs. That a great part of the water passes off into the urine is shown by the great increase of the latter after the use of spirituous liquors, as well as by the peculiar kind of ethereal odor in it, which can be produced though, only by the secretion of a yet unknown secondary product of the alcoholic combustion, which with the water is carried off also by the kidneys. The question now arises: During the general combustion of aldehyde, what is the behavior of the remaining blood in the body, whose metamorphosis into carbonic acid and water are so retarded? The retarding of oxidation affects principally the sugar. If this is not consumed, it must finally either be accumulated in the blood, or be ejected as such (diabetes) with the excrement, or it must undergo some other kind of transformation. We cannot imagine an accumulation of sugar to remain for any length of time in the blood; the author never found it in the urine of any animals experimented upon, it must undergo consequently some other transformation. In relation to the appearance of milituria,the author begged attention to the fact, that many had declared that diabetes was frequently found among drunkards, and considered it possible that, in such cases, it was induced by the above mentioned causes.

The further metamorphosis of sugar is, however, most important. If it is not tranformed into carbonic acid and water, it must change into fat, to which probably the excess of oxygen breathed, is applied. Herein doubtless lies the cause of the excessive formation of fat among drunkards.

To what extent the transformation of the nitrogenous substances suffers from the superior oxidation of aldehyde in the blood is unknown; it may, however, be analogous to what has been previously stated in regard to sugar—the retarding of the metamorphosis, or what is more probable, a change in the nature of the constituents. The principal action of aldehyde upon the blood is generally, therefore, the withdrawal of its oxygen. Its existence is, however, not always indicated by this, but by the rapid action of the processes; the alcohol rapidly oxidizing into aldehyde, and this more readily than other substances, withdrawing rapidly a large amount of oxygen. This probably soon becomes balanced by the increased respiration from the necessity for oxygen; as was observed by the author in his experiments and as is known in life. During intoxication purer air is requisite; therefore in cold climates or in winter a larger amount of alcohol can be taken than in warm climates or in summer. It seems therefore that the rapid absorption of oxygen performs here another part. Those bodies which act in the same or somewhat similar manner, which either absorb oxygen rapidly, or impede its access to other substances, exhibit similar actions. The breathing of carbonic acid and of hydrogen gas produces insensibility and sleep. The intoxicating effect of protoxide of nitrogen is known. The action of opium, on the contrary, can not be explained in the same manner. In opposition to these substances, bodies though of like or similar combination, which are incapable of being oxidized, show no action analogous to that of alcohol.

Of the alcohols, the æthyl, methyl and amyl-alcohols alone act similarly, possessing a like affinity for oxygen. The

æthal, a kind of fatty substance, which oxidizes slowly, acts on the contrary, indifferently on the organism, as the author proves by experiments. Acetic acid as above shown is also wanting in that intoxicating principle, though it must finally be removed from the system by further oxidation. It is therefore not improbable that the rapid withdrawal or the great want of oxygen is essentially the fundamental cause of *most* if not all the phenomena of intoxication. The trembling, the transient paralysis of the lower extremities—which we know to be the case with men, and which were not wanting in any of the author's experiments, cannot therefore depend immediately upon the chemical action of alcohol nor their disappearance upon the inhalation of much oxygen during intoxication, which was observed by the author in his experiments upon birds and dogs. Death, in consequence of the so called alcoholic poison, must occur, agreeably to the above, when with the presence of a great amount of aldehyde in the blood, there exists not at the same time a sufficient amount of oxygen to transform it into acetic acid.

To establish the phenomena that occur after *long continued use of alcohol*, the author instituted several experiments upon dogs. In order to obviate the difficult application of the stomach pump—and to prevent the fluid from getting into the windpipe from the restlessness of the animal—he used gelatin-capsules which contained each 5 grmms. of absolute alcohol. To a dog two years old, rather thin, and of small species, were daily given 15 grmms. of absolute alcohol, which was followed regularly by intoxication. Emaciation and weakness in the hinder limbs, occurred. Death in seventeen days and fourteen hours after the last dose. The blood contained many carbonates and much sugar. To a setter one year old, and well fed, first small, then large doses of alcohol (from 5 to 15 grmms.) until intoxication occurred, were given. Here also appeared weakness in the hinder limbs—emaciation not very great. Death in forty-three days and eight hours after the last dose. A weak odor of aldehyde from the inner organs and blood. The latter contained much sugar. To a small dog, corn brandy was daily given for seventy-two days, until intoxication occurred. The animal became rapidly emaciated, to which a cancerous tumor in the glands of the breast probably contributed. The symptoms of weakness in the hinder limbs were also here apparent. The blood contained no sugar. To another young dog, corn brandy was given for thirty-two days. The same symptoms occurred. The blood contained much sugar. To a large setter, corn brandy was given daily for ninety-three days, from two to three spoonfuls. Upon this he became very fat, and was well, when on the above named day he was accidentally killed. The blood contained much sugar. There was much fat on the under skin of the cellular tissue, otherwise no anatomical change.

The anatomical dissection in the cases of sudden death by alcohol presents no change of any organ, of the brain or its membranes—in the lungs only were found a spurious œdema. Emaciation was shown in long continued habits of intoxication; in the brain though, no change, not even that thickening of the membranes and dropsy in the ventricles, quoted by Huss. In two cases there was blennorrhœa of the external auditory canals. The stomach and its membranes were, in opposition to others' account, always normal. Only in one case where but little amount of brandy had been taken was much fat found. In conclusion, the author abridged his obtained results, as follows:

1. Alcohol undergoes in the system continued combustion, of which the intermediate products are found in the blood.
2. Intoxication is dependent upon the existence of aldehyde in the blood.
3. The action of aldehyde upon the blood is the rapid withdrawal of its oxygen.
4. Therefore the combustion of other substances, consequently their metamorphoses, are prevented or retarded.—*Schmidt's Jahrbuch, October.*

